# Supplementary material for: Pulse of inflammatory proteins in the pregnant uterus of European polecats (Mustela putorius) leading to the time of implantation
Source: R Soc Open Sci. 2017 Mar 22;4(3):161085. doi: 10.1098/rsos.161085 (PMC5383852; doi:10.1098/rsos.161085)
Supplement: Figure S1. Changes in European polecat uterine secretory proteins with time after mating. This figure shows the changes in uterine secreted protein profiles from all the animals used in the study - Figures 1 and 2 of the main text show only a selection of these. [file rsos161085supp1.pdf]

Royal Society Open Science

Pulse of inflammatory proteins in the pregnant uterus of European polecats (*Mustela putorius*) leading to the time of implantation

Heli Lindeberg[a], Richard J.S. Burchmore[b], and Malcolm W. Kennedy[c]

[a] Natural Resources Institute Finland (Luke), Green Technology, Halolantie 31 A, FIN-71750 Maaninka, Finland. [b] Institute of Infection, Immunity and Inflammation, and Glasgow Polyomics, College of Medical, Veterinary and Life Sciences, University of Glasgow, Garscube Campus, Glasgow G61 1QH, Scotland, UK. [c] Institute of Biodiversity, Animal Health and Comparative Medicine, and the Institute of Molecular, Cell and Systems Biology, Graham Kerr Building, College of Medical, Veterinary and Life Sciences, University of Glasgow, Glasgow G12 8QQ, Scotland, UK

Author for correspondence: Malcolm Kennedy (malcolm.kennedy@glasgow.ac.uk)

Supplementary information

Figure S1. Changes in European polecat uterine secretory proteins with time after mating. In this species, implantation usually occurs at about 12 days after mating and gestation lasts for approximately 42 days. Protein SDS-PAGE of all the samples collected periodically from days 4 to 14 after mating. Animals sampled on days 4 and 14 were subsequently found to be non-pregnant. Sample volumes applied to each track of the gel were identical from the original uterine flushes. The resulting band intensity of the strong band at approximately 65 kDa (serum albumin) was subsequently used to adjust sample loading for the protein gels shown in Figures 1 and 2 of the main text. The samples subsequently used for the analyses shown in Figures 1 and 2 are indicated by underlinings. M – Marker/calibration proteins with relative mobilities (M_r) indicated in kilodaltons (kDa). 5-12% gradient gel run under non-reducing conditions, stained with Coomassie Blue.

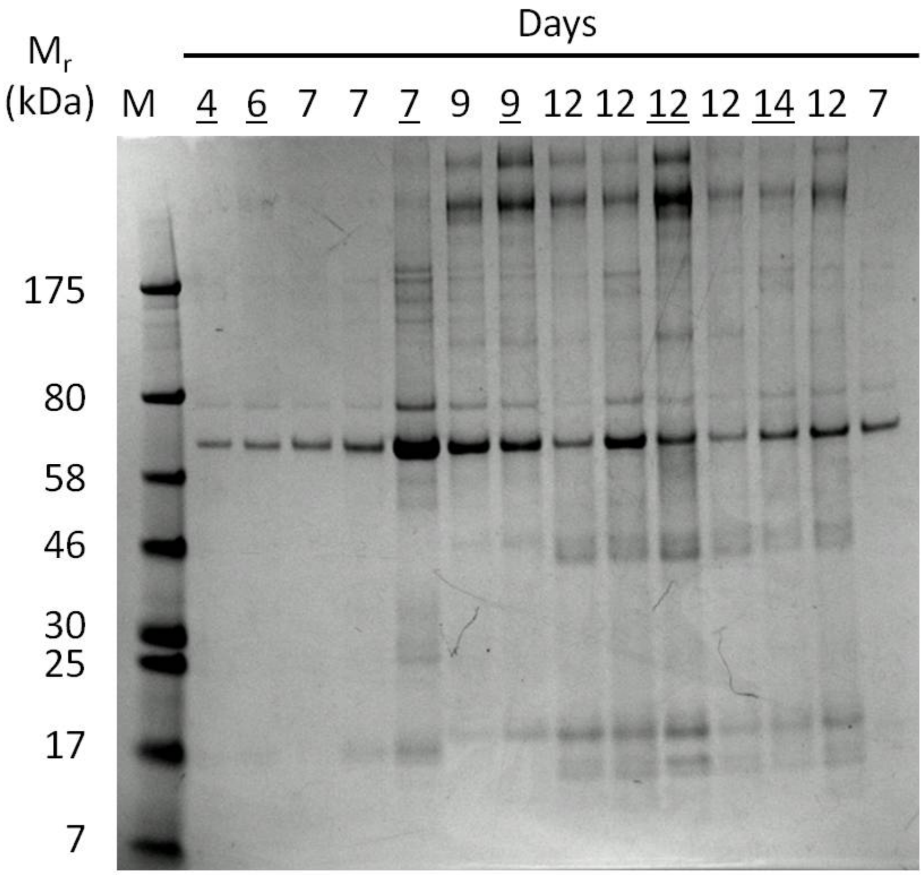

Days
M_r
(kDa)
M
4 6 7 7 7 9 9 12 12 12 12 14 12 7
175
80
58
46
30
25
17
7
